# Exploring needs, perceptions, and preferences towards exercise video among overweight individuals - a qualitative study

**DOI:** 10.12688/f1000research.150772.4

**Published:** 2025-06-16

**Authors:** Shishira K B, K Vaishali, Rajagopal Kadavigere, Suresh Sukumar, Shivashankara K. N., Samuel A. Pullinger, Tulasiram Bommasamudram, Praveen Hoogar

**Affiliations:** 1 Department of Physiotherapy, Manipal College of Health Professions, Manipal Academy of Higher Education, Manipal, Karnataka, 576104, India; 2 Department of Radiodiagnosis and Imaging, Kasturba Medical College, Manipal Academy of Higher Education, Manipal, Karnataka, 576104, India; 3 Department of Medical Imaging Technology, Manipal College of Health Professions, Manipal Academy of Higher Education, Manipal, Karnataka, 576104, India; 4 Department of Medicine, Kasturba Medical College (KMC), Manipal Academy of Higher Education, Manipal, Karnataka, 576104, India; 5 Sport Science Department, Inspire Institute of Sport, Vidyanagar, Dist. Bellary, 583275, India; 6 Department of Exercise and Sports Science, Manipal College of Health Professions, Manipal Academy of Higher Education, Manipal, Karnataka, 576104, India; 7 School of Social Science, The Apollo University, Chittoor, Andhra Pradesh, 517127, India

**Keywords:** Exercise-video, Interview, Need, Overweight, Perception, Preference, Thematic-analysis

## Abstract

**Background:**

The number of overweight people (BMI 25–29.9kg.m ^-2^) in the world is increasing, which increases the risk of health problems and psychological difficulties. To reduce these risks, it is imperative to address unhealthy habits including food and exercise. This qualitative study aimed to explore the needs, perceptions, and preferences of overweight individuals on tailored exercise programs that incorporate educational videos.

**Methodology:**

Forty Individuals between the age of 18-30 years with a BMI of 25-29.9kg.m ^-2^ were included in the study using Purposive sampling from October 2023 to November 2023. Semi-structured in-depth interview was conducted for 45-60 minutes approximately among both active and inactive overweight individuals by a researcher trained in qualitative research. These interviews were audio-recorded and transcribed verbatim. Thematic analysis was performed using inductive and deductive approaches to identify the recurrent patterns, themes, and insights in the transcribed interviews.

**Results:**

8 themes, such as lifestyle factors, exercise explorations, holistic wellbeing and 44 subthemes emerged from the analysis, helping to shed light on the needs, perceptions, and preferences that overweight individuals experience when it comes to their preference for a customized exercise program provided through educational video. The participants wanted personalized regimens that catered to their requirements and levels of fitness.

**Conclusion:**

In summary, the findings emphasize the value of educational videos that are easy to use, visually appealing, and supported by science. These videos should emphasize technique, varying levels of difficulty, and brief sessions.

## Introduction

Overweight individuals: those with a BMI of 25 to 29.9kg.m ^-2 ^are becoming more prevalent nationally and internationally. Non-communicable diseases and being overweight are closely associated, with higher rates of morbidity and mortality. ^1^ People who are overweight are more likely to experience serious health conditions that lower their quality of life. ^2^ Furthermore, being overweight increases the likelihood of developing psychosocial issues such as depression, disordered eating, low self-esteem, and disruptions in body image. ^3^^,^^4^ It is imperative to address unhealthy lifestyle behaviours related to diet, exercise, and coping mechanisms in overweight populations since they exacerbate these disorders. ^5^

Regular exercise is essential for reducing the hazards related to being overweight. ^6^ The World Health Organization (WHO) recommends that adults engage in at least 150 minutes of moderate-intensity aerobic physical activity or 75 minutes of vigorous-intensity activity per week, along with muscle-strengthening activities on two or more days. ^7^ Even with a wealth of evidence showing the health advantages of consistent physical activity, more than half of Indians do not exercise to the recommended levels. ^8^^,^^9^ According to recent studies, approximately 54% of Indians are insufficiently active. ^8^^,^^10^ While many studies consistently report high levels of physical inactivity among Indians, some findings suggest regional variations and inconsistent trends. These discrepancies highlight the need for more region- specific and demographic-specific investigations into physical activity levels. ^9^^,^^11^ One of the main causes of this deficit is frequently stated to be a lack of time. ^12^ The development of an exercise program is significantly influenced by the participants’ feelings and behaviours, which enable us to understand their perception of the program better. ^13^ Although individual variables are important, it is commonly forgotten that social, community and environmental factors also impact physical activity levels. ^14^^,^^15^ Even with a plethora of intervention tools designed to encourage physical exercise, the ongoing prevalence of inactivity highlights the need for more potent approaches. ^16^^–^^18^ Individuals who are overweight or obese frequently encounter physical constraints, diminished self-esteem and social obstacles. ^19^ Understanding the unique requirements, attitudes, exercise perceptions and preferences of overweight people are essential to design focused and successful interventions that deal with the underlying reasons for sedentary behaviour. ^20^ Research suggests that adults, especially younger individuals, often prefer flexible and self-directed exercise formats, such as video-based programs, due to time constraints, varying fitness levels, and a desire for autonomy in their routines. ^21^^–^^24^ An exercise video is the preferred mode, which provides the freedom to modify timing, intensity and exercise type to accommodate individual preferences. By adapting these videos, individuals who are overweight can overcome their barriers to physical activity and develop sustainable, healthy habits. ^25^^,^^26^ The insights acquired from an understanding can be employed in developing individualized exercise video programs that promote inclusivity and improve adherence within this demographic. A noteworthy development in efficacy enhancement has been the provision of individually catered information through interventions. ^27^^–^^29^

It is critical to look into the reasons behind these populations’ low levels of physical activity as well as their preferences for exercise and impediments. Empirical studies regularly demonstrate robust associations between exercise motivation, exercise-related obstacles, personal preferences, and true levels of physical activity. ^30^^–^^32^ As such, it is essential to perform a needs assessment prior to developing an exercise video program tailored for persons who are overweight. Designing interventions that are more likely to boost exercise involvement can benefit from a greater understanding of their experiences.

As a result, the purpose of this study is to explore the current needs, perceptions, and preferences related to exercise among overweight individuals, providing insights into the elements that effectively influence exercise participation.

## Methods

### Study design and recruitment

This qualitative study involves need analysis for the exercise video among overweight individuals through an in-depth interview. The ethical approval was obtained from the Kasturba Medical College and Kasturba Hospital Institutional Ethics Committee, Manipal Academy of Higher Education on 31 ^st^ August 2023 (IEC1: 96/2023). The study adhered to the principles outlined by the declaration of Helsinki. This study followed the standard reporting guidelines of COREQ. ^33^ A purposive sampling was used to recruit study participants. Participants were included in the study if they were 18 to 30 years old and categorized as Overweight with a BMI between 25-29.9kg.m ^-2^ according to WHO classification. We employed open recruitment strategies to ensure adequate representation within the target population by disseminating posters and flyers on the college campus, and social media platforms to reach a broader audience. Pre-participation health screening was determined using the American College of Sports Medicine (ACSM) screening algorithm and the Physical Activity Readiness Questionnaire Plus (PAR Q +). Medical clearance from a physician or other qualified health care provider was recommended if indicated during screening. Individuals with any reported or confirmed comorbidities were excluded from participation. Eligible individuals were required to be inactive or active based on the Global Physical Activity Questionnaire (GPAQ), free from comorbidities such as diabetes, hypertension, CVD, neurological, and musculoskeletal disorders. Additionally, participants needed to be able to verbally communicate in English and express a willingness to participate in the study Eligible participants were informed about the objectives and study procedure, and those who agreed to participate signed the written informed consent. The identity of the individuals has been removed to maintain confidentiality. The recruitment was carried out from October 2023 to November 2023.

### Data collection

A semi-structured face-to-face in-depth interview was conducted among 40 exercising and non-exercising participants by SKB, trained in qualitative research methodology . The sample size of 40 participants was determined based on the study’s objectives and the purposive sampling approach. It was designed to ensure data saturation while allowing for comparisons between two groups. It provided an opportunity to explore variability in contextual elements such as age, fitness levels, and activity patterns. The interview process was guided by an open-ended questionnaire allowing the participants to express their views freely. Through literature reviews and discussions, an interview guide was developed. It was then validated and suitably modified following the advice provided by the experts prior to the interviews. The guide included questions regarding their exercise routine, motivations, barriers to exercise, need for the exercise video, and requirements in the video (refer to the Data set for the interview guide). Interviews were carried out over multiple sessions. Participants were interviewed at scheduled appointments to ensure convenience. The interview process was conducted for 45-60 minutes in a quiet room with only the researcher and the participant. These interviews were audio-recorded on a smartphone and transcribed verbatim. Data triangulation was performed, comprising qualitative interviews with overweight participants, observation of the interactions between participants and the interviewer, and recorded materials. This improved the validity of the qualitative study.

### Analysis

The Content Validity Index (I-CVI and S-CVI) was used to analyze the interview guide’s content validity. Data was analysed manually using inductive and deductive approaches. The manual data analysis procedure consisted of some defined processes. Initially, in-depth interview verbatim transcripts were carefully recorded, and saved electronically, and a physical copy was made for the convenience of analysis. Following multiple readings of the transcripts, two team members (SKB and KV) independently coded the material. Disagreements that occurred during the coding process were resolved by consensus and discussion. Significant statements matching the study’s goals were identified and highlighted as keywords during the first coding step. These keywords were subsequently arranged using axial coding. Keywords with similar meanings were categorized and further combined to form broad themes (selective coding).

## Results

### Participant characteristics

A detailed summary of the 40 participants’ characteristics is given in Table 1 of this qualitative study. The age of the participants spanned between 20 to 30 years, encompassing both male 20 and female 20 individuals with their BMIs varying between 25 and 29.9kg.m ^-2^. Based on their answers to the GPAQ, 20 of the participants were found to be moderately active (> 600 MET per week), and the other 20 were classed as inactive (<600 MET per week). The participants’ educational backgrounds ranged from undergraduate to Ph.D. level qualifications. Only three of the participants were employed while the majority, comprising thirty-seven individuals were students.

**Table 1.** Demographic details of participants.

No	Characteristics	Total
1	Age	
	20-25	27
	26-30	13
2	Gender	
	Male	20
	Female	20
3	BMI	
	25-27	20
	27-29.9	20
4	Activity level (GPAQ)	
	In active (< 600MET/Week)	20
	Moderately active (> 600 MET/Week)	20
5	Education level	
	Undergraduate	14
	Postgraduate	20
	PhD	06
6	Occupation	
	Working	03
	Student	37
7	Marital status	
	Married	36
	Unmarried	04

### Content analysis

The analysis of the in-depth interview yielded 8 themes and 44 subthemes which are described in Table 2.

**Table 2.** Content Analysis of Interview.

Themes	
**1. Lifestyle factors**	
Eating habits	Stress eating Diet
Activity behaviour	Long sitting Mix Exercising Playing
Exercise routine followed	Strength Cardio
Sitting time	5-6 hours 6-7 hours
Enjoyable activities	Dancing Aerobics Badminton Body weight
Incorporate activity	Stairs Walking
**2. Exercise expectations**	
Mental health	Stress relieving Improve concentration. Overcome anxiety
Appearance	Weight loss Muscle tone
Fitness	Improve strength Improve endurance Flexibility
Physical well being	Prevent disease Health risk Healthy lifestyle
Necessity of exercise	Maintain healthy lifestyle Maintain body Prevent disease Confidence
Specific area to target	Arms Abdomen Hips Thighs
Feeling of exercise	
Positive	Happy Satisfying
Negative	Boring Body-conscious
Time commitment to exercise	10-15 minutes, 4-5 days/week
**3. Exercise exploration**	
**Obstacles**	
Personal	Lacks Self-discipline Yearns for Company Body consciousness Fatigue Difficult to exercise after college Injury or menstrual cycle Lacks knowledge of exercise
Environment	Unpleasant weather Commuting to the gym Dedicated space Social prejudice
Opportunity	Lack of time Cost of the gym Lack of knowledge about exercise
**Facilitators**	
Personal	Improve mood Weight management Desire for good health Restricted wardrobe
Environment	Support from parents and friends
Opportunity	Good access Space for exercise
**Major concerns**	Flexibility of muscles Consistency Initiation of the exercise Lack of proper information about exercise (FITT)
**4. Preference of exercise resource**	
Mode	Videos Personal training
Advantages	Easy to follow Convenient User friendly Timesaving Money saving
Disadvantages	Not reliable information Unrealistic recommendations Lacks monitoring
Previous experience	Positive- someone with me, motivational content, animations, visual feedback, short, accessible Negative- lacks information very fast, difficult, lacks scientific evidence, moderate quality, demotivating, branding, advertisement in between exercise videos.
**5. Requirements in the video**	
Techniques	Proper forms or positions Repetitions Sets Breaks
Levels	Easy to harder Easy to perform
Duration	Short duration (10-15min) Not more than 30 min
Instruction mode	Audio and video Captions Muscle involved Experienced instructor, energetic instructor
Exercise routine	Flexible routine
Exercise variety	Exercise recruiting all body parts Progression Tailor-made
Preferred exercise	Equipment free
Music	High intense, beats
**6. Appealing factors**	
Model	Active and fit Similar age group
Approach	Encouraging Friendly Easy to understand
Quality	Good lighting Bright light Clear sound Good video quality
Background	Empty space without distraction Light coloured wall Greenery Based on exercise intensity
Duration	Short duration videos Advertisement free
Presentation	Clarity in communication Immersive colours Different planes
Professionalism	Moderate to high
**7. Holistic well being**	
Nutrition	Calorie intake Diet Water intake
Safety precautions	Attire and shoes to wear Ventilation Non-slippery flooring Do’s and don’ts Monitoring the exhaustion
Exercise benefits	Health benefits of exercise Muscle being recruited Hormonal influence
Mode of delivery	At the end or beginning Disclaimer Separate video
Motivation	Weekly call Exercise done video Logbook Text messages
**8. Media preference**	
Version	YouTube links Downloadable links
Video size	Less internet consumption
Delivery mode	WhatsApp YouTube Email

### Theme 1- Lifestyle factors

Lifestyle factors were illuminated by participants, shedding light on both dietary patterns and exercise routines.

Regarding eating habits, participants (n=11) described that they exhibited stressful eating behaviour during challenging times. However, on the other days (non-stressful periods) they (n=22) follow a disciplined and health-conscious diet.

*“I try to reduce the amount of food I eat; I don’t eat Sweets”.*

*“When I take Stress during exams I just simply eat even If I’m not hungry, I go and buy something and eat”.*

Participants provided insights into their activity patterns, with many (n=33) reporting extended periods of sitting ranging from 5 to 6 hours or 6 to 7 hours daily. The activity behaviours varied, encompassing a combination of sedentary and active pursuits. Some individuals (n=14) adhered to a regular exercise regimen, incorporating either strength or cardio routines or both, while others (n=6) mentioned consistent engagement in activities such as dancing, aerobics, badminton, and body weight exercises, highlighting the incorporation of enjoyable activities into their physical routines.

*“My college work would be, uh, between nine to five, uh, so out of that, uh, maximum of three to four hours in a day maximum uh, would be, uh, my class hours. So, except for that, most of the time I would be sitting”.*

*“I go to the gym regularly 6 days a week and I focus more on weight training and resistance training and around half an hour of cardio in the gym.”*

*“Around 6, 6 15 I’ll go play uh, till 8, 8 30. I mean, uh, obviously I won’t play continuously. Uh, so by the time I leave, uh, badminton, it’ll be around 8, 8 30”.*

### Theme 2- Exercise expectations

The participants expressed exercise expectations regarding mental health, appearance, fitness, and overall well-being while also emphasizing the necessity of exercise for preventing illnesses and building confidence. Their desired exercise regimen involved dedicating 10-15 minutes for 4-5 days per week, focusing on specific areas such as the arms, abdomen, hips, and thighs. While acknowledging the positive impact of exercise on their mood and satisfaction, some participants (n=12) noted potential drawbacks, mentioning feelings of boredom over time and heightened self-consciousness when exercising in the presence of others.

*“Exercise is necessary because as I said, some amount of appearance related concerns, future health risks, plus some amount of, um, mental health related as well”.*

*“So, I personally feel that it makes me feel like I’ve achieved something. Even if I have done a small workout”.*

*“I feel bored. It’s like, playing is much more exciting to me. Like playing or dancing is more exciting to me rather than exercising”.*

### Theme 3- Exercise exploration

The exploration of participants’ exercise experiences revealed a spectrum of obstacles and facilitators influencing their pursuit of physical activity. These challenges were classified into personal factors, environmental factors, and opportunities.

Personal factors comprised aspects like self-discipline, the need for company during exercise, body consciousness, fatigue, challenges in working out after college hours, lack of knowledge, and concerns related to injury.

*“I require some company who can, you know, motivate me to move. Of course, being alone, I won’t”.*

Notably, a significant number of female participants (n=10) identified the menstrual cycle as a major obstacle to regular exercise.

*“I would say, um, cycles, one of the barriers, I guess I can say that during that week, I wouldn’t want to but then, then there’s a break of a one good week and then again you have to put yourself through”.*

Environmental factors played a pivotal role, with adverse weather conditions, difficulties in commuting to the gym, and a lack of dedicated exercise space emerging as significant barriers.

*“I do not after coming here only I understood it. I don’t feel very well with humidity because I sweat a lot”.*

“ *Rainy days actually will affect my activity and feel very lazy. So the weather will make us feel very lazy, so those times It’ll be like kind of difficult for me to go to the gym and do”.*

Social prejudice, particularly the perceived restrictions on females exercising in gyms, was also highlighted (n= 2).

*“My mom doesn’t allow me to go exercise in the gym otherwise, I would just do it in the gym”.*

Various opportunities acted as impediments to participants (n=32) to their regular exercise, including time constraints, the cost of gym memberships, and insufficient knowledge about exercise routines.

“ *It’s obviously easy to join a gym or so, but then gym yeah, again, you need to prioritize the time for that and then a lot of the gyms these days are pretty expensive, so it might not fit into our budgets to go”.*

On the flip side, facilitators to exercise encompassed personal motivations, environmental support, and available opportunities that helped participants overcome these hurdles. Personal motivations included post-exercise mood improvement, weight management goals, and the desire for overall good health.

“ *I’m always striving to be better, that is also there. There’s that motivation to always be better. So, I’m not always satisfied with what I am right now. So that’s one drive or one motivation for me to keep going to achieve something even better”.*

Interestingly, some female participants (n=5) noted that a limited wardrobe served as a compelling motivation to engage in exercise.

*“Not just appearance and I think multiple I mean, apart from, uh, trying to, uh, reduce by exercises I guess certain adaptations, like, I mean, not being able to wear a certain type of clothes, I think that’s something that even motivated to further work out”.*

Support from parents and friends, coupled with easy accessibility and dedicated exercise space, were identified as additional facilitators (n= 19).

Key concerns related to exercise were articulated as challenges in maintaining flexibility, and consistency, initiating exercise routines, and a lack of accurate information about various exercises by experts.

*“Consistency because, yeah, few days after starting to exercise. It’s very hard. You have to force yourself. I remember three years back when I started exercising, I was like blaming myself. But why did you gain weight? It is then I did this two-week challenge, so I had a challenge. So, I was like, OK, anyways I started. I will continue. But yeah, exercising consistently is purely very difficult. You must force yourself”.*

### Theme 4- Preference of exercise resource

Participants (n=28) expressed a clear preference for utilizing video content as their primary exercise resource, with some also indicating a preference for personal training. They highlighted the advantages of videos, citing their ease of follow, user-friendly nature, time and cost-effectiveness, and overall convenience. Additionally, participants (n=36) shared positive experiences with past exercise videos, appreciating the sense of exercising with a companion, motivational content, animated visuals, immediate feedback, short durations, and easy accessibility compared to other resource forms.

*“Videos because we can see the emotions. Like letters and pamphlets, we can read that, but there won’t be such an emotion. So, when we see a video, for example, I’m not exercising, so they are actually, we can feel that how they work out, emotions, feelings, everything. So that will make us do”.*

On the downside, they (n=31) noted drawbacks such as insufficient information on techniques, unrealistic recommendations, excessively fast paced and challenging routines that could demotivate, and a lack of monitoring and proper scientific evidence. Some participants (n= 3) also expressed dissatisfaction with branding and advertisements associated with the videos.

*“Most of the videos that we see on the Internet don’t take into that much of an account of everything else. They just say a set of exercises and they say that it would help you lose weight within these many days, or like which is unrealistic amount of time, so they usually what we see is unrealistic set of goals and time plan”.*

### Theme 5- Requirements in the video

This segment was meticulously crafted, focusing on various aspects such as exercise techniques, difficulty levels, duration, instructional modes, diverse exercise routines, preferred activities, and the role of music in the video. All participants (n=40) expressed a strong desire for detailed guidance, including proper form, specific numbers of repetitions and sets, and designated breaks. They emphasized the importance of a range of difficulty levels, spanning from easy to challenging, yet manageable within a shorter duration (n=25) of 10-15 minutes.

*“I would want the complete breakdown of the Exercise. Then the uh…. number of reps for muscle building, muscle loss, weight building, weight loss. Then the timings, the breaks in between everything to be proper and easy to comprehend”.*

Moreover, participants (n=26) sought a flexible exercise routine that engaged all body parts, tailored to individual abilities, and visually demonstrated with a progression guide. They (n=20) specifically requested clear captions and voice-over instructions provided by an experienced instructor, outlining the muscles involved.

*“A combination of audio and video like uh…. at each key phase and explaining what is happening there would be better”.*

Additionally, some participants (n=29) expressed a preference for high-intensity music to be incorporated into the video.

*“Definitely, uh…… I think music and exercises is one good combination. Even when I’m walking actually, I have my earphones on all the time, helps me with at least getting that rhythm. If it is for a video uh…. I think something without lyrics something that gives or pumps yourself”.*

### Theme 6- Appealing factors

The factors that resonated with participants included their preferences for models, the approachability of the presenter, video quality, background settings, and the duration of exercise content. They (n=21) expressed a preference for an active and fit individual of a similar age, serving as the demonstrator for all exercises in the video. This person should adopt an encouraging approach that is easy to understand.

*“Somebody who looks fit because sometimes not looking fit then the person who is following the person might just feel what is the whole point of doing this. So, uh… Somebody who looks fit and of the same age, who is confident about what they are doing, what they are saying”.*

Participants (n=28) specifically favoured videos presented with bright lighting, clear sound, and visual clarity against a light-coloured background in a distraction-free space. They (n=22) emphasized the importance of the video exhibiting a moderate to high level of professionalism and featuring immersive colours.

*“Some plain background or dark background with good lighting which is standardized because even mind appreciates light, and uh… the participant performing the exercises and his movement pattern is clearly visible”.*

Additionally, participants (n=25) highlighted their desire for the video to be of short duration and free from advertisements.

*Uh, short duration videos, maybe, uh 10 minutes, uh, or max to max 15 minutes. Uh, uh, I would suggest keep it short. Uh, and much, uh, focusing, uh, that should be more than enough.*

### Theme 7- Holistic wellbeing

This explored the participant’s considerations beyond exercise and physical activity encompassing aspects of nutrition, safety precautions, exercise benefits, mode of delivery, and motivational strategies.

All participants (n=40) expressed a preference for information covering calorie and water intake, suitable attire and footwear for exercise, the exercise environment’s features such as ventilation and non-slip flooring, guidelines for the dos and don’ts of exercises, and methods for monitoring exhaustion.

*“The main thing is shoes, clothing, and uh…. everything related to ventilation, hydration, and nutrition. My perspective I will be happy”.*

Moreover, they sought insights into the health benefits of exercises and the specific muscles engaged during different activities. Particularly, female participants (n=6) indicated a keen interest in understanding the hormonal influences of exercise.

*“And also, hormonal influences if you’re talking about female population those are also, uh, major parts. So, educating them about these things are very much important. Rather than telling them exercise, telling how they can actually get into those programs is much important”.*

Regarding motivation, participants (n=23) indicated a preference for weekly communication, either through calls or texts, from fitness experts. Additionally, they expressed a desire to track their progress through videos or logbooks to enhance their commitment to regular exercise.

“ *Yaa actually, if we talk to the person only, then only we’ll be coming to know that what exactly is happening”.*

Their preference was for this information to be presented either at the beginning of a video or as a disclaimer at the end.

### Theme 8- Media preferences

The featured videos are intended (n= 34) to be shared with participants through their preferred platforms, such as YouTube, WhatsApp, or email. The content will be presented as a link, which requires minimal internet usage and facilitates easy downloading.

*“Like, let’s say if I’m outside, Uh, I don’t have that much, uh, data, on the phone So that time I would think, uh, it would be better if I had a downloaded video”.*

*“Thing is YouTube link would be nice. Email and WhatsApp are my preferred modes”.*

## Discussions

The objective of this qualitative study was to explore the current needs, perceptions, and preferences related to exercise among overweight individuals. The in-depth interview analysis offers insightful information about the perceptions and experiences of overweight people regarding a range of topics, including lifestyle factors, expectations for exercise, exercise exploration, preferred exercise resources, requirements in videos, appealing factors, holistic well-being, and media preferences. These themes make a substantial contribution to our understanding of the complex interactions among social, environmental, and personal factors that influence exercise behaviours.

### Lifestyle factors

This theme clarified the complex interrelationships between lifestyle elements, including food habits and exercise regimens. One of the main conclusions showed that individuals’ eating habits followed a dual pattern that was influenced by stress. Those who were going through difficult situations tended to eat poorly, but during less stressful times, they ate more healthily and with discipline. This dynamic interaction between food decisions and emotional well-being highlights how differently people react to stress and its impact on lifestyle. In addition, the analysis of exercise regimens and sitting duration offered a thorough picture of the lifestyles of the participants, placing them in the range of sedentary to moderately active. Likewise, research conducted by O’Neil et al. (2012) and Cha et al. (2015) also documented widespread unhealthy lifestyle patterns. ^34^^,^^35^ This conclusion is also consistent with the results of a study by Kushner et al. (2010) that showed how prevalent harmful lifestyle patterns are—including poor food, exercise habits, and coping mechanisms among overweight or obese. The study found a correlation between these patterns and advancing age, and that they intensified with rising BMI. ^5^ The incorporation of these findings into the broader context of existing research points to the need for a more complex understanding of the variables driving unhealthy lifestyle choices, particularly in populations dealing with weight-related issues. Identifying these trends becomes an essential first step toward developing focused interventions. According to Kushner et al (2010), interventions that target both emotional health and certain lifestyle decisions may be more successful in encouraging healthy behaviours in people who struggle with being overweight or obese. ^5^ This emphasizes how important it is to take a comprehensive approach when creating therapies that are specific to the problems that unhealthy lifestyle patterns create.

### Exercise expectations

The discussion on participants’ expectations for exercise demonstrates the diverse nature of perspectives on physical activity. People approach exercise with a variety of objectives in mind, highlighting the importance of customized routines. The recognition of allocating time to target body parts emphasizes a tactical approach to fitness, rejecting a one-size-fits-all paradigm. Positive recognition of exercise’s impact on mood aligns with the literature on mental health benefits. ^36^^,^^37^ However, it also highlights potential negative effects including boredom and self-consciousness, emphasizing the need for diversity and psychological considerations. This pattern of persistent negativity aligns with findings by Sikes et al. (2019), who highlighted boredom as the predominant factor contributing to youth’s physical inactivity. ^38^ The various expectations that participants have about their physical and mental well-being, appearance, and fitness highlight the necessity of inclusive exercise programs. Overall, the results support customized and holistic approaches to satisfy a range of needs and encourage sustained adherence to healthy lifestyle choices.

### Exercise exploration

A thorough examination of the exercise experiences of the participants in our study revealed a wide variety of barriers and enablers, which we divided into three categories: opportunistic, environmental, and personal variables. In terms of personal variables, difficulties were particularly associated with discipline and body consciousness. These were mentioned by participants as the key obstacles to adhering to a regular exercise schedule. Social prejudice and unfavourable weather patterns emerged as major obstacles on the environmental side. These results resonate with the systematic analysis conducted by Peng et al. (2023) among young adult women, emphasizing the multifaceted and interconnected nature of the challenges they encounter such as time, social support, body image, and cultural beauty standards. ^39^ Enhancing physical activity among women necessitates addressing not only individual behaviours but also creating opportunities at the organizational, community, and policymaker levels. These elements add to a thorough comprehension of the challenges people have when following an exercise program.

Nevertheless, our study’s identification of individual motivations, social support, and accessible opportunities as facilitators suggests possible intervention and support strategies to increase regular exercise participation. By focusing on these facilitators, interventions can be customized to help individuals overcome obstacles and encourage long-term physical activity. Concurrently, variables such as enhanced physical attractiveness, health advantages, and favourable psychological impacts were recognized as motivators, indicating a wider agreement on significant influences. The results of this study are consistent with previous research since other studies have also brought attention to perceived motivations such as maintaining or improving physical appearance, health benefits, and togetherness, while also focusing on perceived obstacles such as dislike of exercise, a lack of motivation, a lack of support, and time constraints. ^40^^,^^41^

Further guiding the creation of focused exercise modules in the researched populations’ emphasis on flexibility, consistency, initiation, and the accessibility of reliable exercise information. Adopting strategies that target these specific concerns can improve the efficacy of programs intended to encourage physical activity participation.

In summary, the thorough investigation of the variables affecting exercise regimens, as demonstrated by our research, and corroborated by the body of literature, offers insightful design information for customized interventions. By comprehending the intricacies of personal experiences and addressing both obstacles and enablers, we may aid in the advancement of more potent approaches designed to encourage consistent exercise engagement across a variety of demographics.

### Preference of exercise resource

In the context of fitness and weight control programs, professionals face the difficulty of creating interventions that promote health and induce behavioural change without creating a feeling of social rejection. This project highlights the real-world difficulties in creating workout programs specifically for overweight people. The importance of widely accessible and user-friendly platforms is emphasized in Theme 4, which highlights the general preference for video content as the primary source for exercise. Purcell (2010) noted a trend among younger groups toward video content consumption. ^42^ Vandelanotte et al. (2011) discovered that a video-based physical activity intervention was well-received by potential users, indicating its feasibility for development. This tendency could be attributed to the influence of social norms, as younger individuals usually establish developing internet trends that later gain traction across broader groups. ^43^ Although participants find video materials engaging and convenient, they have concerns over their dependability and oversight. This dual viewpoint points out places where video-based workout tools need to be improved. The acknowledgment of video content’s advantages indicates participants’ engagement and motivation for fitness. Prioritizing accessibility and user-friendliness aligns with the modern desire for seamless experiences. However, concerns about monitoring and reliability highlight specific drawbacks and highlight the need for a more participatory and responsible experience. This insight draws attention to limitations in video-based fitness regimens and offers suggestions for improvement. In broad terms, this knowledge is essential for creating successful online workout regimens. Understanding user preferences and concerns allows fitness platforms to balance the convenience of video content with the need for reliability and monitoring. Features like progress tracking and engagement can improve the effectiveness and user happiness of online workout programs. To better address a variety of needs and encourage an active and healthy lifestyle, digital fitness solutions must be refined through ongoing user-developer conversation.

### Requirements in the video

This section emphasizes the importance of making exercise videos specifically tailored for overweight people, with a focus on creating a supporting atmosphere with specially trained instructors. Fostering a positive and inclusive atmosphere can be enhanced by addressing specific needs and concerns. The exploration of participants’ preferences emphasizes the requirement to provide precise instructions on exercise methods, levels of difficulty, duration, and instructional modalities. The findings align with previous research by Manaf et al. (2021) and Othman et al. (2022). These studies indicate a preference among participants for a low to moderate-intensity structured exercise regimen that offers flexibility along with manageable duration and frequency. These findings indicate a need for participant’s favoured workouts to accommodate varying schedules and environments ^40^^,^^41^ Participants in our study expressed a desire for flexible routines engaging all body parts, accompanied by music, emphasizing holistic and adaptable workout experiences that consider enjoyment and diversity as factors for maintaining motivation. Bian et al. (1989) highlighted the importance of such characteristics, finding that participants favoured exercise regimens tailored for overweight individuals or beginners, emphasizing the need for instructors with expertise, a non-judgmental attitude, along with proper pacing. ^13^ Citing research such as Wankel et al., (1985) highlights the valuable insights that may be gained from fitness programs by highlighting the possible influence of strong social support. ^44^ This realization raises the possibility that overcoming variations in individual self-motivation requires a strong sense of community and support. Finally, this area explores the preferences of the participants and provides insightful information to develop a comprehensive approach that is effective and specific to the needs of overweight individuals by emphasizing support, clear guidance, flexibility, and social components.

### Appealing factors and holistic well-being

Our study delved into factors influencing participant engagement, particularly focusing on the presentation and aesthetics of exercise videos. Preferences for relatable models, an encouraging approach, and professionally produced, distraction-free shorter videos highlight the importance of creating visually appealing and immersive experiences. These insights align with Vandelanotte et al.’s 2011 study on computer-tailored interventions using online video messages to promote physical activity laying the groundwork for understanding participant preferences and openness to innovative approaches. ^43^ It is widely believed that internet-delivered interventions, particularly those with high interactivity and user appeal, promote increased participant engagement and retention. Furthermore, such interventions may offer increased and sustained effectiveness over the long term ^18^^,^^45^

Additionally, participants expressed a desire for comprehensive information, including details on nutrition, safety precautions, exercise benefits, and motivational strategies. Solbrig et al., (2017) in his study concluded that individuals expressed a desire for motivational support for losing weight and increasing physical activity. He stated that there exists a mismatch between available public resources and the individuals desired health information regarding lifestyle information. ^46^ This aligns with a holistic perspective recognizing the interconnectedness of physical and mental well-being, suggesting interventions should address both aspects.

Furthermore, information on hormonal influences and regular communication from fitness experts address participant’s broader health concerns, emphasizing the need for comprehensive support structures within intervention programs to ensure adherence. Our study contributes valuable insights for designing holistic interventions beyond exercise guidance to address various factors influencing users.

### Media preferences

The media platforms that participants prefer to use, such as email, WhatsApp, and YouTube, show a wide variety of consumption patterns. While WhatsApp and email reflect a need for immediate communication and easy access to information, YouTube suggests a preference for exercise material that is primarily based on videos. The focus on connections with low internet consumption needs draws attention to the necessity of content optimization for accessibility, taking participants’ possible connectivity issues into account. Comprehending these inclinations is essential to customizing the content of exercises and maximizing the effectiveness of health promotion programs. By aligning dissemination strategies with favoured platforms, health professionals can maximize reach and impact. These results are consistent with research showing the feasibility of video-tailored interventions. ^43^^,^^47^^,^^48^ Such interventions are viable both in terms of user willingness to engage and technological viability. This is supported by the fact that most internet users have access to infrastructure that supports video downloads. According to Vandelanotte et al.’s (2011) findings, exercise interventions that consider participant preferences and include extensive support systems may enhance user engagement and promote long-term adherence. ^43^ The understanding also highlights the importance of providing relevant, approachable content that fits participants’ circumstances to increase engagement and encourage adherence to recommended exercise regimens.

### Strengths and limitations

The methodology employed in this study was equally as important as the results. Our one-on-one interviewing approach created a comfortable and secure environment for individuals to express their ideas. Everyone might express himself without feeling compelled to by others. To the best of our knowledge, our study is the first to examine the needs, perceptions, and preferences of overweight individuals about exercise videos. The limitation of the study was it involved purposive sampling and the findings may not be easily applicable to broader more general populations beyond the ones studied. The generalizability of findings to wider populations, particularly those from diverse cultural, socioeconomic, or regional backgrounds, was limited. Despite this limitation, purposive sampling was suitable for the study’s aim, as it ensured contributions from individuals who conformed to the desired user profile and were most likely to benefit from the intervention. The study provides a valuable framework to develop tailored exercise interventions, which can guide future modifications for varied populations.

## Conclusion

In conclusion, our qualitative research not only dives into the multiple elements that influence exercise behaviours among overweight individuals but also provide a thorough comprehension of their preferences for instructive exercise videos. The findings from this analysis provide a good framework for developing customized interventions that consider the complex interaction of individual experiences, preferences, and problems.

This knowledge, together with the findings from our investigations into several themes such as lifestyle determinants, exercise expectations, and media preferences, guides the development of programs that go beyond a standard approach. Integrating personalized exercise recommendations enhances participation rates and fosters long-term behavioral change. Developing guidelines to encourage the production and dissemination of targeted exercise content may further support obesity prevention and management. By acknowledging the unique needs of overweight individuals, we aim to create effective, engaging, and accessible educational exercise videos. Future studies should incorporate diverse perspectives to address these limitations and further explore the long-term impact of tailored exercise programs.

## Authors contributions

SKB and KV conceptualized and designed the study protocol. Data collection was conducted by SKB with verification by KV. SKB and PH was involved in data curation. SKB prepared the preliminary draft of the manuscript. Scientific content and relevance were critically curated by SKB, VK, RKV, SS, SKN, SP and TB. The final manuscript version received approval from all authors before journal submission. Each author fulfilled the criteria set forth by ICMJE for authorship, contributed significantly to the manuscript, and critically reviewed and approved the final draft.

## Ethics and consent

The ethical approval was obtained from the Kasturba Medical College and Kasturba Hospital Institutional Ethics Committee, Manipal Academy of Higher Education on 31st August 2023 (IEC1: 96/2023). The study adhered to the principles outlined by the declaration of Helsinki ( https://www.wma.net/policies-post/wma-declaration-of-helsinki-ethical-principles-for-medical-research-involving-human-subjects/). Eligible participants were informed about the objectives and study procedure, and those who agreed to participate signed the written informed consent.

## Data Availability

The study’s underlying and extended data: OSF: “Exploring needs, perceptions, and preferences towards exercise video among overweight individuals - a qualitative study”; 2024. Available from: https://doi.org/10.17605/OSF.IO/XSTBC. ^49^ Data are available under the terms of the **CC0 1.0 Universal** .
