# Supplementary material for: Exploring needs, perceptions, and preferences towards exercise video among overweight individuals - a qualitative study
Source: F1000Res. 2025 Jun 16;13:998. Originally published 2024 Sep 2. [Version 4] doi: 10.12688/f1000research.150772.4 (PMC12134721; doi:10.12688/f1000research.150772.4)
Supplement: Peer review history

# Reviewer report: Reviewer response for version 3

**DOI:** 10.5256/f1000research.180273.r378056

**Published:** 2025-06-03

**Authors:** Adriyan Pramono

**Affiliations:** 1 Diponegoro University, Semarang, Indonesia

**Summary:**

 This manuscript offers a valuable qualitative investigation into the needs, perceptions, and preferences of overweight young adults (BMI 25–29.9 kg·m⁻², aged 18–30 years) regarding the design and delivery of tailored exercise programs via educational videos. The authors successfully identify key thematic areas (including lifestyle factors, exploration of exercise modalities, and holistic well-being) that underscore the participants’ desire for personalized, visually engaging, and evidence-based content. The study effectively highlights the importance of concise, user-friendly videos that clearly demonstrate exercise techniques and offer varying levels of difficulty. Nonetheless, several improvements are recommended to enhance the clarity and overall rigor of the manuscript.

**Introduction:**

 In the following statement *“An exercise video is the preferred mode, which provides the freedom to modify timing, intensity and exercise type to accommodate individual preferences”*, it is recommended to support this claim with additional context regarding age-related preferences for exercise delivery modes. Doing so would reinforce the relevance of this delivery method for the target population (in this case are adults) and strengthen the connection between existing literature and the current study's focus.

**Discussion:**

 Following the limitations section, it is advisable for the authors to reflect on the potential impact of these limitations on the study's validity and generalizability. A brief discussion on whether the limitations meaningfully affect the manuscript's contribution to the field would help readers assess the study’s utility as a reference for future research.

**Further Comments:**

 To ensure the manuscript meets established standards for high-quality qualitative research, it is recommended that the authors consider aligning their work with recognized reporting guidelines. Specifically, the use of the *Standards for Reporting Qualitative Research (SRQR)* checklist (available at https://doi.org/10.1097/acm.0000000000000388) would improve transparency and replicability.

Is the work clearly and accurately presented and does it cite the current literature?

Yes

If applicable, is the statistical analysis and its interpretation appropriate?

Yes

Are all the source data underlying the results available to ensure full reproducibility?

Yes

Is the study design appropriate and is the work technically sound?

Yes

Are the conclusions drawn adequately supported by the results?

Yes

Are sufficient details of methods and analysis provided to allow replication by others?

Yes

Reviewer Expertise:

Nutrition (specifically about overweight)

I confirm that I have read this submission and believe that I have an appropriate level of expertise to confirm that it is of an acceptable scientific standard, however I have significant reservations, as outlined above.

# Reviewer report: Reviewer response for version 2

**DOI:** 10.5256/f1000research.178289.r374517

**Published:** 2025-04-03

**Authors:** Waqar M Naqvi

**Affiliations:** 1 Datta Meghe Institute of Higher Education and Research, Wardha, India

INTRODUCTION:

 The authors are encouraged to briefly discuss the importance of exercise videos as a platform specifically designed for individuals who are overweight. Justifying this focus would improve the study's relevance and clarity.

 METHODOLOGY:

 The authors should specify whether data triangulation was performed and, if so, address it specifically in the methodology section.

 Further clarification regarding the coding process is needed, particularly a thorough description of how disagreements among team members were handled and resolved.

 RESULTS:

 It is suggested that one or two major themes that emerged from the results be included in the abstract to enhance its insightfulness.

 CONCLUSION:

 The conclusion could be improved by highlighting how the results may influence public health policy or guide the development of future exercise interventions.

Is the work clearly and accurately presented and does it cite the current literature?

Yes

If applicable, is the statistical analysis and its interpretation appropriate?

Yes

Are all the source data underlying the results available to ensure full reproducibility?

Yes

Is the study design appropriate and is the work technically sound?

Yes

Are the conclusions drawn adequately supported by the results?

Yes

Are sufficient details of methods and analysis provided to allow replication by others?

Yes

Reviewer Expertise:

Tele - rehabilitation, Gamification, AR,  VR, Ai in health, Serious Gaming

I confirm that I have read this submission and believe that I have an appropriate level of expertise to confirm that it is of an acceptable scientific standard.

# Response

**Published:** 2025-04-04

**Authors:** Shishira K B

**Affiliations:** Department of Physiotherapy, Manipal Academy of Higher Education, Manipal, Karnataka, India

Dr Waqar M Naqwi,

 Thank you for your valuable feedback and for accepting our article. We appreciate your insightful suggestions, which have helped us enhance the clarity and relevance of our manuscript.

**INTRODUCTION: **The authors are encouraged to briefly discuss the importance of exercise videos as a platform specifically designed for individuals who are overweight. Justifying this focus would improve the study's relevance and clarity.

 We appreciate your suggestion to discuss the importance of exercise videos as a platform specifically designed for individuals who are overweight. In response, we have now incorporated a discussion on the challenges faced by overweight individuals and how exercise videos can help overcome these barriers.

**METHODOLOGY: **The authors should specify whether data triangulation was performed and, if so, address it specifically in the methodology section. 

 Thanks for highlighting. We have now specified whether data triangulation was performed and  addressed it in the methodology section, as suggested.

 Further clarification regarding the coding process is needed, particularly a thorough description of how disagreements among team members were handled and resolved.

 Disagreements among the team members were resolved through discussion and consensus, this has now been added in the manuscript. Thank you

**RESULTS: **It is suggested that one or two major themes that emerged from the results be included in the abstract to enhance its insightfulness.

 Thank you for your suggestion to include major themes in the abstract to enhance its insightfulness. We have now incorporated key themes from the results section into the abstract 

**CONCLUSION: **The conclusion could be improved by highlighting how the results may influence public health policy or guide the development of future exercise interventions. 

 Thank you for your guidance on strengthening the conclusion by discussing the influence of result on public health policy. We have made the changes as per your suggestion in the conclusion.

# Reviewer report: Reviewer response for version 1

**DOI:** 10.5256/f1000research.165369.r343442

**Published:** 2024-12-11

**Authors:** Adi Pranoto

**Affiliations:** 1 Universitas Negeri Surabaya, Surabaya, Indonesia

The authors have clearly stated that the study aims to explore the needs, perceptions, and preferences of overweight individuals on tailored exercise programs that incorporate educational videos. The paper is well-written, easy to follow and adds merit to the vital role of educational videos that are easy to use, visually appealing, and supported by science. These videos should emphasize technique, varying levels of difficulty, and brief sessions. Given this approach, this work can enhance future attempts in similar research area. However, I have highlighted a few suggestions and concerns in my specific comments section (below) that need to be addressed before considering whether this work should be indexed or not.

 ABSTRACT

 - Exercise-video, Interview, Need, Overweight, Perception, Preference, Thematic-analysis. I suggest reducing keywords by taking the 5 most important keywords

 INTRODUCTION

 - “Even with a wealth of evidence showing the health advantages of consistent physical activity, more than half of Indians do not exercise to the recommended levels. 7,8” I highlight the following statement that researchers say more than half of Indians do not exercise at the recommended level. Therefore, I suggest researchers add the data on how many Indians in total are not exercising at the recommended levels. Apart from that, researchers can also add the recommended duration of exercise, such as recommendations from WHO ( https://www.who.int/news-room/fact-sheets/detail/physical-activity).

 - I suggest researchers add, perhaps this can be supported by the results of previous research with inconsistent results.

 MATERIALS AND METHODS

 - How do you recruit potential participants? Forty individuals aged between 18-30 years with a BMI of 25-29.9kg.m-2 who were involved in the study using purposive sampling from October 2023 to November 2023 were obtained in what way? Do you do open recruitment or in certain communities? Please provide a detailed explanation in the methods section.

 - How do researchers obtain data about the medical records of each participant, so that researchers can ensure that participants are free from comorbidities such as diabetes, hypertension, CVD, neurological and musculoskeletal disorders?

 - In the data collection process, were the 40 participants involved interviewed at one time or were there differences in time?

 - Has the questionnaire used in this study been tested for validity and reliability? If so, can I see the results of the analysis?

 - In the Analysis subsection, researchers can add validity and reliability tests.

 - Please describe how the sample size is calculated?

 RESULTS

 - The results are presented well and in detail.

 DISCUSSION

 - The discussion section should present paragraphs to improve reading conditions. It should start with the objective of the study, followed by the main findings, and afterward relating these with the literature. It is also important to develop limitations and suggestions for future research at the end of this section.

 CONCLUSION

 - Add some recommendations for future research and add strong reasons to support the study.

 REFERENCES

 - Please adjust to the F1000Research reference writing style as there are still many discrepancies found.

 GENERAL COMMENTS

 - Pay attention to english grammar, syntax rules and punctuation all over the manuscript.

Is the work clearly and accurately presented and does it cite the current literature?

Yes

If applicable, is the statistical analysis and its interpretation appropriate?

I cannot comment. A qualified statistician is required.

Are all the source data underlying the results available to ensure full reproducibility?

Yes

Is the study design appropriate and is the work technically sound?

Yes

Are the conclusions drawn adequately supported by the results?

Yes

Are sufficient details of methods and analysis provided to allow replication by others?

Partly

Reviewer Expertise:

NA

I confirm that I have read this submission and believe that I have an appropriate level of expertise to confirm that it is of an acceptable scientific standard, however I have significant reservations, as outlined above.

# Response

**Published:** 2025-02-14

**Authors:** Shishira K B

**Affiliations:** Department of Physiotherapy, Manipal Academy of Higher Education, Manipal, Karnataka, India

Thank you for reviewing the paper. 

 ABSTRACT

 - Exercise-video, Interview, Need, Overweight, Perception, Preference, Thematic-analysis. I suggest reducing keywords by taking the 5 most important keywords

**Response:** Thank you, we have reduced the key words to the 5 most important words.

 INTRODUCTION

 - “Even with a wealth of evidence showing the health advantages of consistent physical activity, more than half of Indians do not exercise to the recommended levels. 7,8” I highlight the following statement that researchers say more than half of Indians do not exercise at the recommended level. Therefore, I suggest researchers add the data on how many Indians in total are not exercising at the recommended levels. Apart from that, researchers can also add the recommended duration of exercise, such as recommendations from WHO ( https://www.who.int/news-room/fact-sheets/detail/physical-activity).

**Response:** Thank you for highlighting the point. We have added the percentage of Indians that do not exercise.

 - I suggest researchers add, perhaps this can be supported by the results of previous research with inconsistent results.

**Response: **Thanks for your suggestions. The inconsistent results with respect to previous research has been addressed in the introduction section.

 MATERIALS AND METHODS

 - How do you recruit potential participants? Forty individuals aged between 18-30 years with a BMI of 25-29. 9kg.m-2 who were involved in the study using purposive sampling from October 2023 to November 2023 were obtained in what way? Do you do open recruitment or in certain communities? Please provide a detailed explanation in the methods section.

**Response:** Detailed  recruitment of potential participants has now been added in the new version of the manuscript. It’s an open recruitment that we followed in this study.

 - How do researchers obtain data about the medical records of each participant, so that researchers can ensure that participants are free from comorbidities such as diabetes, hypertension, CVD, neurological and musculoskeletal disorders?

**Response:** Participants were screened before recruiting them into the study using screening tools American College of Sports Medicine (ACSM) screening algorithm and the Physical Activity Readiness Questionnaire Plus (PAR Q +). Medical clearance from a physician or other qualified health care provider was recommended if indicated during screening.

 - In the data collection process, were the 40 participants involved interviewed at one time, or were there differences in time?

**Response: **Interviews were carried out over multiple sessions.

 - Has the questionnaire used in this study been tested for validity and reliability? If so, can I see the results of the analysis?

**Response: **Thank you for your feedback. The questionnaire underwent a validation process using the Content Validity Index (CVI) and demonstrated a good CVI score (Clarity- 0.78, Relavence-0.81, Comprehensiveness- 0.82). Based on expert evaluations, modifications were made. Specifically, two questions were removed as they were deemed inappropriate, one question was suggested to be used as a probe rather than a standalone item, one question was revised for better clarity, and an additional question was incorporated based on expert recommendations. The modified version of the questionnaire was subsequently used in the study.

 - In the Analysis subsection, researchers can add validity and reliability tests.

**Response: **Thanks for the feedback. We have added as The Content Validity Index (I-CVI and S-CVI) was used to analyze the interview guide's content validity

 - Please describe how the sample size is calculated?

**Response:** The sample size of 40 participants was determined based on the study’s objectives and the purposive sampling approach. It was designed to ensure data saturation while allowing for comparisons between two groups. It provided an opportunity to explore variability in contextual elements such as age, fitness levels, and activity patterns.

 RESULTS

 - The results are presented well and in detail.

**Response:** Thanks for the compliments.

 DISCUSSION

 - The discussion section should present paragraphs to improve reading conditions. It should start with the objective of the study, followed by the main findings, and afterward relating these with the literature. It is also important to develop limitations and suggestions for future research at the end of this section.

**Response: **Thanks for guiding the discussion format. The suggestion to add objective has been incorporated.

 CONCLUSION

 - Add some recommendations for future research and add strong reasons to support the study.

**Response:** Thank you for the suggestion. Future scope for the study is added in the conclusion.
